# Eco-friendly role of *serratia marcescens* and *pseudomonas fluorescens* in enhancing rice growth and mitigating cadmium toxicity via uptake modulation and antioxidant regulation

**DOI:** 10.1186/s12870-025-06693-6

**Published:** 2025-05-28

**Authors:** Yousef Alhaj Hamoud, Hiba Shaghaleh, Muhammad Hamzah Saleem, Mohammed O. Alshaharni, Mohammed Alqurashi, Seham Sater Alhelaify, Ohud Muslat Alharthy, Eman Fayad, Anshu Rastogi

**Affiliations:** 1 College of Hydrology and Water Recourses, Hohai University, Nanjing, 210098 China; 2 College of Environment, Hohai University, Nanjing, 210098 China; 3 Office of Academic Research, Office of VP for Research & Graduate Studies, Qatar University, Doha, 2713 Qatar; 4 Biology Department, College of Science, King Khalid University, Abha, 61421 Saudi Arabia; 5 Department of Biotechnology, College of Sciences, Taif University, P.O. Box 11099, Taif, 21944 Saudi Arabia; 6 Laboratory of Bioclimatology, Department of Ecology and Environmental Protection, Faculty of Environmental and Mechanical Engineering, Poznan University of Life Sciences, Piatkowska 94, Pozna ´n, 60-649 Poland

**Keywords:** Plant growth promoting-rhizobacteria, Heavy metal accumulation, Cereal crop, Proline, Gas exchange parameters

## Abstract

Plant growth-promoting rhizobacteria (PGPR) offer sustainable means to enhance crop resilience under environmental stress, including heavy metal toxicity. Understanding their role in mitigating such stresses is vital for advancing biotechnological strategies aimed at food security and sustainable agriculture. A pot experiment was conducted to determine the effects of single and/or combined application of different levels [10 and 20 ppm] of *Serratia marcescens* and *Pseudomonas fluorescens* on Cd accumulation, morpho-physio-biochemical attributes of rice (*Oryza sativa* L.) exposed to severe Cd stress [0 (without Cd stress), and 100 µM)]. The research outcomes indicated that elevated levels of Cd stress in the soil significantly (*p* ≤ 0.05) decreased plant growth and biomass, photosynthetic pigments, and gas exchange attributes. However, Cd stress also induced oxidative stress in the plants by increasing malondialdehyde (MDA) and hydrogen peroxide (H_2_O_2_), which also induced increased compounds of various enzymatic and non-enzymatic antioxidants and also the gene expression and sugar content. Furthermore, a significant (*p* ≤ 0.05) increase in proline metabolism, the ascorbate–glutathione (AsA–GSH) cycle were observed. Although, the application of *S*. *marcescens* and *P*. *fluorescens* showed a significant (*p* ≤ 0.05) increase in plant growth and biomass, gas exchange characteristics, enzymatic and non-enzymatic compounds, and their gene expression and also decreased oxidative stress. In addition, the application of *S*. *marcescens* and *P*. *fluorescens* enhanced cellular fractionation and decreased the proline metabolism and AsA–GSH cycle in *O*. *sativa* plants. Research findings, therefore, suggest that the application of *S*. *marcescens* and *P*. *fluorescens* can ameliorate Cd toxicity in *O*. *sativa*, resulting in improved plant growth and composition under metal stress, as depicted by balanced antioxidant defense mechanism.

**Graphical Abstract:**

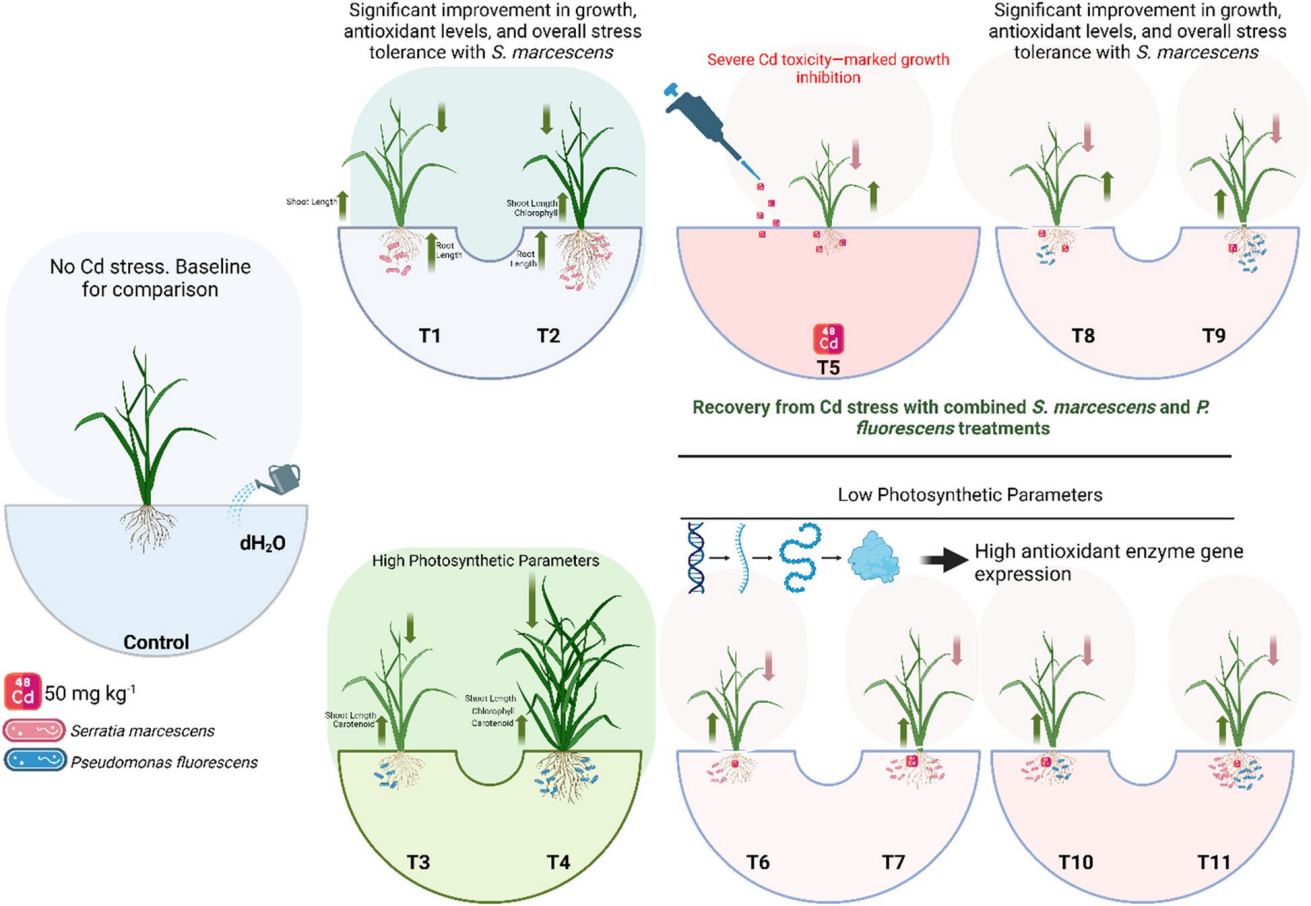

**Supplementary Information:**

The online version contains supplementary material available at 10.1186/s12870-025-06693-6.

## Introduction

Heavy metal accumulation in soils is of great concern in agricultural production due to its adverse effects on food safety and marketability, crop growth due to phytotoxicity, and the environmental health of soil organisms [1]. Heavy metals include cadmium (Cd), lead (Pb), nickel (Ni), cobalt (Co), iron (Fe), zinc (Zn), chromium (Cr), iron (Fe), arsenic (As), silver (Ag) and the platinum group elements [2]. Contamination of agricultural soils with Cd has become one of the most toxic and widespread environmental problems [3]. Photosynthesis, respiration, cell division, water relations, opening and closing of stomata, nitrogen metabolism, and mineral nutrition are the main metabolic processes within the plants, which are negatively affected by Cd stress [1, 4]. Although Cd is toxic for plant growth, it is easily taken by the roots and then transported to the shoots where it can cause retorted growth, stunted root development, reduce branching, alteration in photosynthesis and respiration, diminished nutrient uptake, blocked electron transport chain as well as changed the membrane permeability [5]. Hence, it is immensely required to safeguard plant from Cd toxicity to counter the phytotoxicity and oxidative stress triggered by the uptake of Cd in plants. Rice (*Oryza sativa* L.) is a cereal grain, it is the most widely consumed staple food for a large part of the world’s human population, especially in Asia and Africa. *O. sativa* cultivation is well-suited to countries and regions with low labor costs and high rainfall, as it is labor-intensive to cultivate and requires ample water [6].

As a result, there is an urgent need to develop eco-friendly and effective strategies to mitigate Cd-induced damage in rice cultivation. Among the promising biological approaches, PGPR have attracted considerable attention for their ability to support plant growth under stress conditions [7, 8]. Notably, species such as *S*. *marcescens* and *P*. *fluorescens* have demonstrated potential in promoting plant growth, synthesizing phytohormones, inducing systemic resistance, and reducing heavy metal toxicity through multiple mechanisms [9, 10]. These bacteria not only enhance root development but also produce siderophores, antibiotics, and antioxidant enzymes that enable plants to thrive in adverse environments [11, 12]. Although the individual benefits of PGPR are well-documented, the combined effect of *S. marcescens* and *P. fluorescens* in alleviating Cd toxicity in rice—particularly through the regulation of metal uptake, antioxidant defense systems, and metabolic processes—remains underexplored.

Globalization causes increased in the Cd content in the environment which imposes a critical threat to the food security and production of *O. sativa*. Keeping in the view of importance of *O. sativa* as a cereal crop, we have conducted the present study to investigate the potential of *S*. *marcescens* and *P*. *fluorescens* on *O. sativa* by studying growth and physiological attributes in Cd contaminated soil. Despite the many useful advantages related to the individual applications of *S*. *marcescens* and *P*. *fluorescens*, but the combined application to these growth PGPR still remains unclear in combating the metal stress. Our study hypothesized the individual and combined application of *S*. *marcescens* and *P*. *fluorescens* to *O. sativa* under Cd stress. The results from this study would add to our knowledge about (I) the morphological and photosynthetic efficiency (II) oxidative stress and response of enzymatic and non-enzymatic responses along with their specific gene expression (III) proline metabolism, AsA–GSH cycle, cellular fractionation and Cd uptake in different parts of the plants under the Cd stress with the individual or combined application of *S*. *marcescens* and *P*. *fluorescens*.

## Materials and methods

### Plant growth and experimental setup

**Fig. 1:**
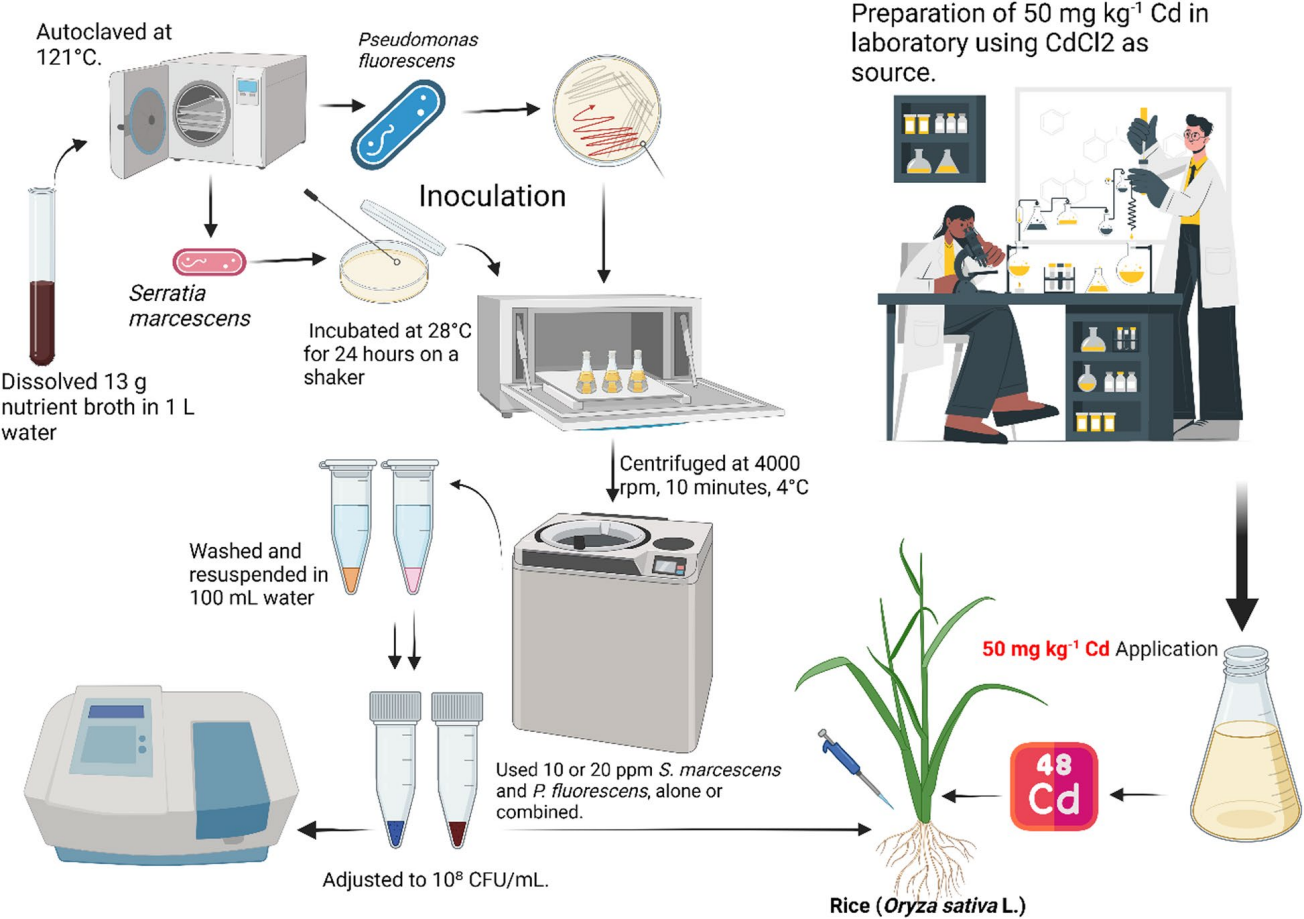
Methodology for PGPR preparation and application in *O*. *sativa* seedlings under Cd stress. The figure illustrates the preparation and application of *S. marcescens* and *P. fluorescens* inoculation at concentrations of 10⁸ CFU/mL, applied via pipetting around seedling roots and soil drenching at 10 or 20 ppm. Cd stress was induced using a 50 mg kg^−1^ CdCl₂ solution. The experimental setup includes individual and combined treatments of PGPR with or without Cd exposure

A pot experiment was conducted in the Department of Biotechnology, College of Sciences, Taif University, P.O. Box 11,099, Taif 21,944, Saudi Arabia, under the glass house. Pots were placed under a glass house environment where they received natural sunlight, day/night humidity (60/70%), and day/night temperature (24/12 °C), respectively. *O. sativa* seeds were taken from the Taif University, P.O. Box 11,099, Taif 21,944, Saudi Arabia. Before sowing, the seeds were carefully washed and sterilized in 0.1% HgCl_2_ solution for 1 min and then washed three times with distilled water. Uncontaminated soil, obtained from the research field of Taif University, P.O. Box 11,099, Taif 21,944, Saudi Arabia, was air-dried and passed through a 2-mm sieve. The physicochemical properties of the soil used for the pot experiment are as follow: pH-6.9, EC-0.9 dS cm^−1^, organic matter 17 g kg^−1^, EK 21 mg kg^−1^, TP 0.17 g kg^−1^ and TN-16 g kg^−1^. After contamination of soil with Cd using cadmium chloride (CdCl_2_) at a level 100 µM in the soil, pots (30-cm-tall * 40-cm-wide) were filled with 10 kg of amended soil and undergone at four different cycles of water equilibrated for 2 months and then air. During the entire experiment, we did not observe any symptoms of waterlogging in *O. sativa*. Application of PGPR was incubated in the growth medium as the method presented by Jaffer et al. [7]. The bacterial isolates were biochemically characterized based on the protocols outlined in"Bergey’s Manual of Determinative Bacteriology"by Holt et al. [13]. The pots used in this study were rotated regularly in order to avoid environmental effects on the plants. A complete randomized design (CRD) with four replications was used. The total duration of experimental treatments was 2 months under controlled conditions. A total of 12 treatments were applied: CK (control, no Cd + *S. marcescens* + *P. fluorescens*), T1 (*S. marcescens*, 10 ppm), T2 (*S. marcescens*, 20 ppm), T3 (*P. fluorescens*, 10 ppm), T4 (*P. fluorescens*, 20 ppm), T5 (Cd, 100 µM), T6 (Cd + *S. marcescens*, 10 ppm), T7 (Cd + *S. marcescens*, 20 ppm), T8 (Cd + *P. fluorescens*, 10 ppm), T9 (Cd + *P. fluorescens*, 20 ppm), T10 (Cd + *S. marcescens* 10 ppm + *P. fluorescens* 10 ppm), and T11 (Cd + *S. marcescens* 20 ppm + *P. fluorescens* 20 ppm). A detailed schematic presentation of the entire methodology is provided in Fig. 1.

### Incubation with PGPR

The effects of *S. marcescens* and *P. fluorescens* on *O. sativa* under Cd stress were evaluated by applying these PGPR under controlled conditions. Both bacterial strains were obtained from a certified microbial culture collection and cultured in nutrient broth (peptone 5.0 g/L, beef extract 3.0 g/L, sodium chloride 5.0 g/L) at 28 °C for 24–48 h with continuous shaking at 150 rpm until an adequate cell density was reached. The cultures were then centrifuged at 5,000 rpm for 10 min, and the bacterial pellets were resuspended in sterile 0.85% sodium chloride (NaCl) solution. The bacterial suspensions were adjusted to a concentration of approximately 1 × 10⁸ colony-forming units per milliliter (CFU/mL), confirmed by measuring the optical density at 600 nm (OD₆₀₀) using a UV–visible spectrophotometer [14]. Corresponding CFU/mL values were determined using standard curves calibrated for each strain [15].

### Plant harvesting and data collection

After two months, remaining seedlings were up rooted and washed gently with the help of distilled water to eliminate the aerial dust and deposition. Functional leaf in each treatment was picked at a rapid growth stage during 09:00–10:30 a.m. The sampled leaves were washed with distilled water, immediately placed in liquid nitrogen, and stored in a freezer at − 80 °C for further analysis. All the harvested plants were divided into two parts i.e. roots and shoots to study different physio-biochemical traits. Leaves from each treatment group were picked for chlorophyll, carotenoid, oxidative stress and antioxidants analysis. Root and shoot lengths were measured straightway after the harvesting by using measuring scale and digital weighting balance to measure fresh biomass. Roots were uprooted and immersed in 20 mM Na_2_EDTA for 15–20 min to remove Cd adhered to the root surfaces. Then, roots were washed thrice with distilled water and finally once with de-ionized water and dried for further analysis. The different parts of the plant (roots and shoots) were oven-dehydrated at 65 °C for 72 h for Cd determination and the total plant dry weight was also measured.

### Chlorophyll pigments and gas exchange attributes determination

For chlorophyll content analysis, 0.1 g of fresh leaf sample was extracted with 8 mL of 95% acetone for 24 h at 4 °C in the dark. The absorbance of the resultant solution was measured by a spectrophotometer (UV-2550; Shimadzu, Kyoto, Japan) at 646.6, 663.6, and 450 nm. The chlorophyll content was then calculated using the standard method described by Arnon [16], ensuring that the final volume of each sample, after adding the supernatant to 85% acetone, was adjusted to 10 mL.

On the same days, gaseous exchange was also measured. Net photosynthesis (P*n*), leaf stomatal conductance (G*s*), transpiration rate (T*s*), and intercellular carbon dioxide concentration (C*i*) were measured from three different plants in each treatment group. Measurements were taken from 9:00 a.m. to 11:00 a.m. to ensure consistent environmental conditions with a clear sky. Rates of leaf P*n*, G*s*, T*s*, and C*i* were measured with an LI-COR gas-exchange system (LI6400; LICOR Biosciences, Lincoln, NE, USA) with a redblue LED light source on the leaf chamber. In the LI-COR cuvette, CO_2_ concentration was set as 380 mmol mol^–1^, and LED light intensity was set at 1000 mmol m^–2^ s^–1^, which is the average saturation intensity for photosynthesis in *O. sativa* [17].

### Analysis of oxidative stress indicators

The degree of lipid peroxidation was evaluated as malondialdehyde (MDA) content. Briefly, 0.1 g of frozen leaves were ground at 4 °C in a mortar with 25 mL of 50 mM phosphate buffer solution (pH 7.8) containing 1% polyethene pyrrole. The homogenate was centrifuged at 10,000 × g at 4 °C for 15 min. The mixtures were heated at 100 °C for 15–30 min and then quickly cooled in an ice bath. The absorbance of the supernatant was recorded by using a spectrophotometer (xMark™ microplate absorbance spectrophotometer; Bio-Rad, United States) at wavelengths of 532, 600 and 450 nm. Lipid peroxidation was expressed as l mol g^−1^ using the following formula: 6.45 (A532 − A600) − 0.56 A450. Lipid peroxidation was measured using a method previously published by Heath and Packer [18].

For hydrogen peroxide (H_2_O_2_) assay, leaf and root samples were homogenized with 50 mM phosphate buffer at pH 6.5. After that, homogenized samples were centrifuge at 6000 × g for 25 min, followed by the addition of H_2_SO_4_ (20% v/v) and again centrifuged at 6000 × g for 15 min. H_2_O_2_ contents were estimated by taking the absorbance at 410 nm and calculations were completed with the help of extinction coefficient (0.28 μmol^−1^ cm^−1^) [19].

### Enzymatic antioxidants determination

To evaluate enzyme activities, fresh leaves (0.5 g) were homogenized in liquid nitrogen and 5 mL of 50 mmol sodium phosphate buffer (pH 7.0), including 0.5 mmol EDTA and 0.15 mol NaCl. The homogenate was centrifuged at 12,000 × g for 10 min at 4 °C, and the supernatant was used for the measurement of superoxidase dismutase (SOD) and peroxidase (POD) activities. SOD activity was assayed in 3 mL reaction mixture containing 50 mM sodium phosphate buffer (pH 7), 56 mM nitro blue tetrazolium, 1.17 mM riboflavin, 10 mM methionine, and 100 μL enzyme extract. Finally, the sample was measured by using a spectrophotometer (xMark™ microplate absorbance spectrophotometer; Bio-Rad). Enzyme activity was measured using a method by Chen and Pan [20] and expressed as U g^−1^ FW.

Peroxidase activity in the leaves and roots was estimated using the method of Sakharov and Ardila [21] using guaiacol as the substrate. A reaction mixture (3 mL) containing 0.05 mL of enzyme extract, 2.75 mL of 50 mM phosphate buffer (pH 7.0), 0.1 mL of 1% H_2_O_2_ and 0.1 mL of 4% guaiacol solution was prepared. Increases in the absorbance at 470 nm because of guaiacol oxidation was recorded for 2 min. One unit of enzyme activity was defined as the amount of the enzyme.

Catalase (CAT) activity was analyzed according to Aebi [22]. The assay mixture (3.0 mL) was comprised of 100 μL enzyme extract, 100 μL H_2_O_2_ (300 mM) and 2.8 mL 50 mM phosphate buffer with 2 mM ETDA (pH 7.0). The CAT activity was measured from the decline in absorbance at 240 nm as a result of H_2_O_2_ loss (ε = 39.4 mM^−1^ cm^−1^).

Ascorbate peroxidase (APX) activity was measured according to Nakano and Asada [19]. The mixture containing 100 μL enzyme extract, 100 μL ascorbate (7.5 mM), 100 μL H_2_O_2_ (300 mM), and 2.7 mL 25 mM potassium phosphate buffer with 2 mM EDTA (pH 7.0) was used for measuring APX activity. The oxidation pattern of ascorbate was estimated from the variations in wavelength at 290 nm (ε = 2.8 mM^−1^ cm^−1^).

Quantitative real-time PCR (RT-qPCR) assay was applied to investigate the expression levels of 4 stress-related genes, including Fe-SOD, POD, CAT and APX. Total RNA was extracted from leaf tissue samples using RNeasy Plant Mini kits (Qiagen, Manchester, UK). The RT-qPCR was performed using an Applied Biosystems 7500 Fast Real-Time PCR System with the following cycling parameters: initial denaturation at 95 °C for 5 min, followed by 40 cycles of denaturation at 95 °C for 15 s, annealing at 58–60 °C (optimized for each primer pair) for 30 s, and extension at 72 °C for 30 s. A melting curve analysis was conducted from 65 °C to 95 °C to confirm the specificity of the amplification. Contaminating DNA was then removed and first-strand cDNAs were prepared using Reverse Transcription kits (Qiagen, Manchester, UK). RT-qPCR analysis was conducted as reported in the protocol of QuantiTect SYBR Green PCR kit (Qiagen, Manchester, UK). Reaction volume and PCR amplification conditions were adjusted as mentioned by El-Esawi et al. [15]. The gene amplifications of Sirhindi et al. [23] of the following genes are given in Table 1S.

### Non-enzymatic compounds and sugar determination

Plant ethanol extracts were prepared for the determination of non-enzymatic antioxidants and some key osmolytes. For this purpose, 50 mg of dry plant material was homogenized with 10 mL ethanol (80%) and filtered through Whatman No. 41 filter paper. The residue was re-extracted with ethanol, and the 2 extracts were pooled together to a final volume of 20 mL. The determination of flavonoids [24], phenolics [25], ascorbic acid [26], anthocyanin [27], and total sugars [28] was performed from the extracts. Fresh leaf material (0.1 g) was mixed thoroughly in 5 mL aqueous sulphosalicylic acid (3%). The mixture was centrifuged at 10,000 × g for 15 min, and an aliquot (1 mL) was poured into a test tube having 1 mL acidic ninhydrin and 1 mL glacial acetic acid. The reaction mixture was first heated at 100 °C for 10 min and then cooled in an ice bath. The reaction mixture was extracted with 4 mL toluene, and test tubes were vortexed for 20 s and cooled. Thereafter, the light absorbance at 520 nm was measured by using a UV–VIS spectrophotometer (Hitachi U-2910, Tokyo, Japan). The free proline content was determined on the basis of the standard curve at 520 nm absorbance and expressed as µmol^−1^ (g FW).

### Determination of proline metabolism

To measure proline concentrations, 0.5 g of shoot tissues were ground in sulfosalicylic acid and then centrifuged, and the supernatant was collected from each sample. The proline concentration in each sample was measured [29]. Specifically, the supernatant from each sample was reacted with acid ninhydrin, and the resulting colorimetric reaction was measured to determine the proline concentration by “UV-1700 pharmaSpec spectrophotometer”.

The ProDH “proline dehydrogenase”, P5 CR “pyrroline-5-carboxylate reductase”, and P5 C “pyrroline-5-carboxylate” were measured using kits provided by Jiangsu Meibiao Biological Technology Co., Ltd. Enzyme activities were accurately measured using these reagent kits, which include all chemicals and related instructions by “UV-1700 pharmaSpec spectrophotometer”.

### Determination of AsA-GSH cycle

Glutathione (GSH), glutathione disulfide (GSSH), DHA (dehydroascorbic acid), and ascorbic acid (AsA) were determined in fresh leaves [30] and were extracted by homogenizing 0.2 g of leaves in TCA and then collecting the supernatant by centrifugation. GSH concentration was measured in a solution including phosphate buffer, supernatant, and DTNB reagent (PBS, pH 7.0) (Singh et al. 2015). The amount of GSH was determined by a spectrophotometer. To measure the AsA content, NaH_2_PO_4_ solution, enzyme extract, distilled water, and 10% TCA were mixed to determine the concentration of AsA in the samples [30]. After a 30-s incubation period, FeCl_3_ solution, H_3_PO_4_, and 2,2′ -dipyridine were added to the reaction mixture. The FeCl_3_ and 2,2′ -dipyridine reacted with the AsA to produce a red-colored complex that can be measured spectrophotometrically at 525 nm. The amount of AsA present in the sample was calculated.

### Determination of cell wall component fractionation

Cell wall isolation was done as reported by Yang et al. [31]. Shoots (4 g) were placed in a mortar and ground with liquid nitrogen. The homogenized samples were transferred to centrifuge tubes and 75% ethanol was added and incubated at 25 °C. The samples were centrifuged. The bottom sediment was further homogenized in 10 mL of each of acetone, chloroform, and methanol (v:v = 1:1) for 30 min each, with shaking at room temperature. The homogenate was centrifuged. The remaining cell wall components were lyophilized until dry sediment was obtained. The lyophilized cell wall components were analyzed for biochemical assays. Subsequently, the separation of the hemicellulose fraction was carried out [31]. Approximately 3 mg CW was mixed with water in an Eppendorf tube. The mixture was boiled for 1 h using a heating block or hot plate set at 100 °C and centrifuged. The above procedure was repeated for duplicate samples. After 12 h, the precipitate was extracted twice with 1 mL of KOH (24%, w/v) at room temperature. After each extraction, centrifugation was done. The hemicellulose concentration was measured at 540 nm absorbance.

Pectin Assay Kit was used to detect pectin. Pectinesterase Assay Kit was used to detect PME activity. The Cellulose Assay Kit was used to detect cellulose concentrations using kits provided by Jiangsu Meibiao Biological Technology Co., Ltd. Enzyme activities were accurately measured using these reagent kits, which include all chemicals and related instructions. DM was calculated using the formula: demethylation degree = 100—DM, where DM is the degree of methylation**.**

### Determination of Cd contents

Finely ground samples were digested with pure HNO_3_ at 190 °C for 45 min (10 min pre-heating, 15 min heating, 20 min cooling) in a microwave oven (Mars 6, CEM Corporation, USA) with the settings described in details by Jezek et al. [32]. Samples were diluted with 2% HNO_3_ and determined by inductively coupled plasma-mass spectroscopy (ICP-MS; Agilent 7700, Agilent Technologies Inc., USA).

### Statistical analysis

All the data in this study were given as arithmetic means analyzed by Statistix 8.1. The values are the means of four replications, one-way analysis of variance (ANOVA) was used (*p* ≤ 0.05) process to evaluate the influence of photo technology on contaminated soil remediation. The mean between different treatments was analyzed using Fisher’s highest significant difference (HSD) test. Graphical representation was conducted using Origin-2017.

## Results

### Impact of individual/or combinatorial application of PGPR on plant growth and photosynthetic pigments under Cd stress

**Fig. 2:**
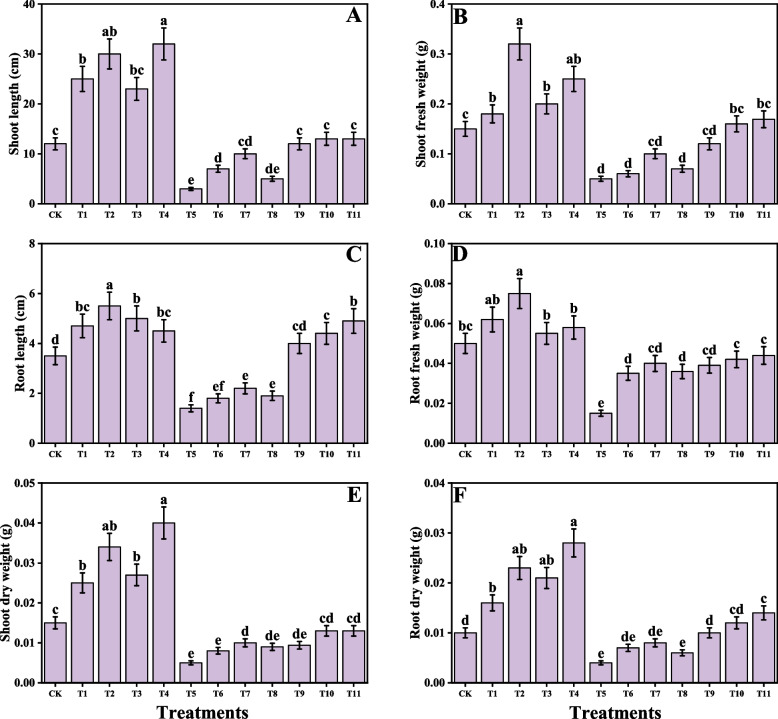
Effect of alone and/or combinatorial application of *Serratia marcescens and Pseudomonas fluorescens* on growth related attributes i.e., (**A**) shoot length, (**B**) shoot fresh weight, (**C**) root length, (**D**) root fresh weight, (**E**) shoot dry weight, and (**F**) root dry weight of rice (*Oryza sativa* L.) grown under the cadmium stress (100 µM) in the soil. Values in the figures indicate just one harvest of four replications. Mean ± SD (*n* = 4). One-way ANOVA was performed and mean differences were tested by HSD (*p* ≤ 0.05). Different lowercase letters on the error bars indicate significant differences between the treatments. The treatments abbreviations are as following: CK: control (no Cd + *S. marcescens* + *P. fluorescens*), T1: *S. marcescens* (10 ppm), T2: *S. marcescens* (20 ppm), T3: *P. fluorescens* (10 ppm), T4: *P. fluorescens* (20 ppm), T5: Cd (100 μM), T6: Cd (100 μM) + *S. marcescens* (10 ppm), T7: Cd (100 μM) + *S. marcescens* (20 ppm), T8: Cd (100 μM) + *P. fluorescens* (10 ppm), T9: Cd (100 μM) + *P. fluorescens* (20 ppm), T10: Cd (100 μM) + *S. marcescens* (10 ppm) + *P. fluorescens* (10 ppm), and T11: Cd (100 μM) + *S. marcescens* (20 ppm) *P. fluorescens* (20 ppm)

**Fig. 3:**
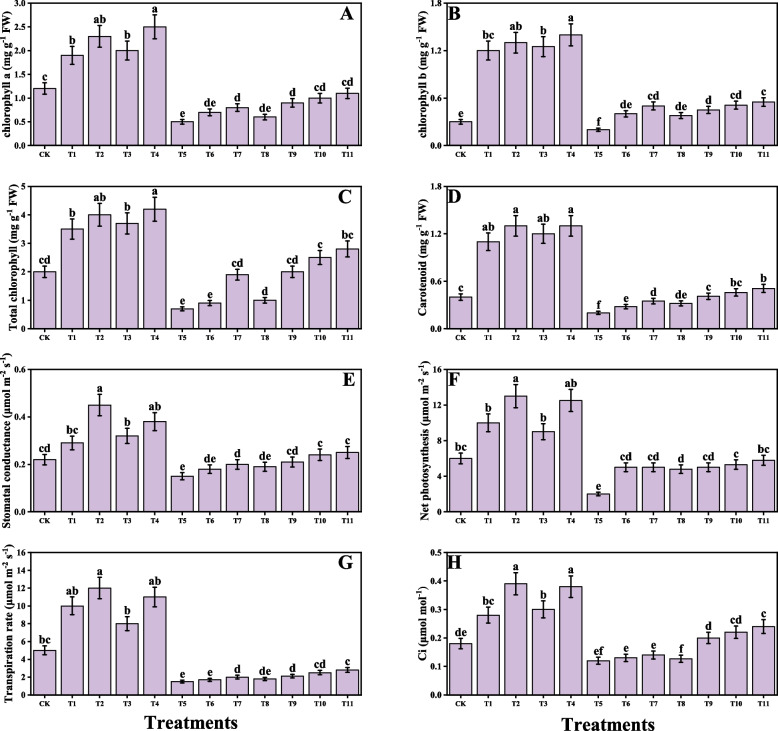
Effect of alone and/or combinatorial application of *Serratia marcescens and Pseudomonas fluorescens* on chlorophyll and gas exchange: chlorophyll-a (**A**), chlorophyll-b (**B**), Total chlorophyll (**C**), carotenoid (**D**), stomatal conductance (**E**), net photosynthesis (**F**), transpiration rate (**G**) and intercellular CO_2_ (**H**) of rice (*Oryza sativa* L.) grown under the cadmium stress (100 µM) in the soil. Values in the figures indicate just one harvest of four replications. Mean ± SD (*n* = 4). One-way ANOVA was performed and mean differences were tested by HSD (*p* ≤ 0.05). Different lowercase letters on the error bars indicate significant differences between the treatments. The treatments abbreviations are as following: CK: control (no Cd + *S. marcescens* + *P. fluorescens*), T1: *S. marcescens* (10 ppm), T2: *S. marcescens* (20 ppm), T3: *P. fluorescens* (10 ppm), T4: *P. fluorescens* (20 ppm), T5: Cd (100 μM), T6: Cd (100 μM) + *S. marcescens* (10 ppm), T7: Cd (100 μM) + *S. marcescens* (20 ppm), T8: Cd (100 μM) + *P. fluorescens* (10 ppm), T9: Cd (100 μM) + *P. fluorescens* (20 ppm), T10: Cd (100 μM) + *S. marcescens* (10 ppm) + *P. fluorescens* (10 ppm), and T11: Cd (100 μM) + *S. marcescens* (20 ppm) *P. fluorescens* (20 ppm)

In the present study, various growth parameters and photosynthetic pigments and also the gas exchange parameters in *O. sativa* under the Cd stress with the application of *S. marcescens* and *P. fluorescens* were measured. Growth and biomasses and also the photosynthetic pigments are presented in Fig. 2 and gas exchange characteristics are presented in Fig. 3. According to the given results, Cd stress caused a significant toxicity in *O. sativa* and decreases shoot length, shoot fresh weight, root length, root fresh weight, shoot dry weight, root dry weight, chlorophyll-a, chlorophyll-b, total chlorophyll, carotenoid content, stomatal conductance, net photosynthesis, transpiration rate, and intercellular CO₂ concentration compared to control. However, application of *S. marcescens* and *P. fluorescens* caused increased in shoot length, shoot fresh weight, root length, root fresh weight, shoot dry weight, root dry weight, chlorophyll-a, chlorophyll-b, total chlorophyll, carotenoid content, stomatal conductance, net photosynthesis, transpiration rate, and intercellular CO₂ compared to the plants which were grown in the control. The application of *S. marcescens* and *P. fluorescens*, when applied to the plants which were not treated with Cd stress, there was a significant increase in shoot length, shoot fresh weight, root length, root fresh weight, shoot dry weight, root dry weight, chlorophyll-a, chlorophyll-b, total chlorophyll, carotenoid content, stomatal conductance, net photosynthesis, transpiration rate, and intercellular CO₂ was observed in the plants compared to those which were not treated with the application of AMF and PGPR. Although the combined application of *S. marcescens* and *P. fluorescens* induced higher growth and biomass and gas exchange attributes, compared to the individual applications of *S. marcescens* and *P. fluorescens*.

### Impact of individual/or combinatorial application of PGPR on oxidative stress, antioxidant capacity and Cd uptake under Cd stress

**Fig. 4:**
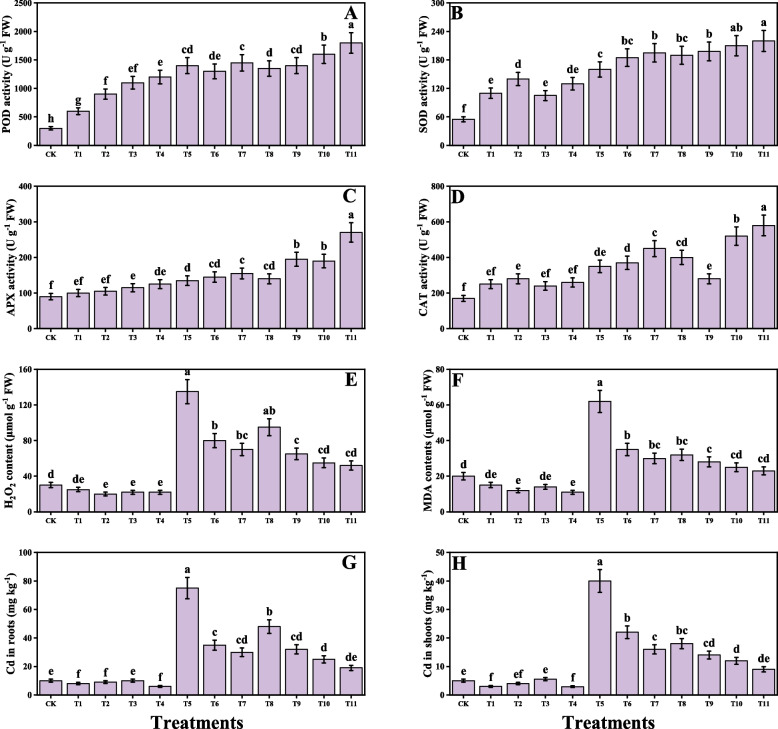
Effect of alone and/or combinatorial application of *Serratia marcescens and Pseudomonas fluorescens* on enzymatic antioxidants and oxidative stress biomarkers i.e., peroxidase (**A**), superoxidase dismutase (**B**), ascorbate peroxidase (**C**), catalase (**D**), hydrogen peroxide (**E**) malondialdehyde (**F**) Cd concentration in the roots (**G**), and Cd concentration in the shoots (**H**) of rice (*Oryza sativa* L.) grown under the cadmium stress (100 µM) in the soil. Values in the figures indicate just one harvest of four replications. Mean ± SD (*n* = 4). One-way ANOVA was performed and mean differences were tested by HSD (*p* ≤ 0.05). Different lowercase letters on the error bars indicate significant differences between the treatments. The treatments abbreviations are as following: CK: control (no Cd + *S. marcescens* + *P. fluorescens*), T1: *S. marcescens* (10 ppm), T2: *S. marcescens* (20 ppm), T3: *P. fluorescens* (10 ppm), T4: *P. fluorescens* (20 ppm), T5: Cd (100 μM), T6: Cd (100 μM) + *S. marcescens* (10 ppm), T7: Cd (100 μM) + *S. marcescens* (20 ppm), T8: Cd (100 μM) + *P. fluorescens* (10 ppm), T9: Cd (100 μM) + *P. fluorescens* (20 ppm), T10: Cd (100 μM) + *S. marcescens* (10 ppm) + *P. fluorescens* (10 ppm), and T11: Cd (100 μM) + *S. marcescens* (20 ppm) *P. fluorescens* (20 ppm)

**Fig. 5:**
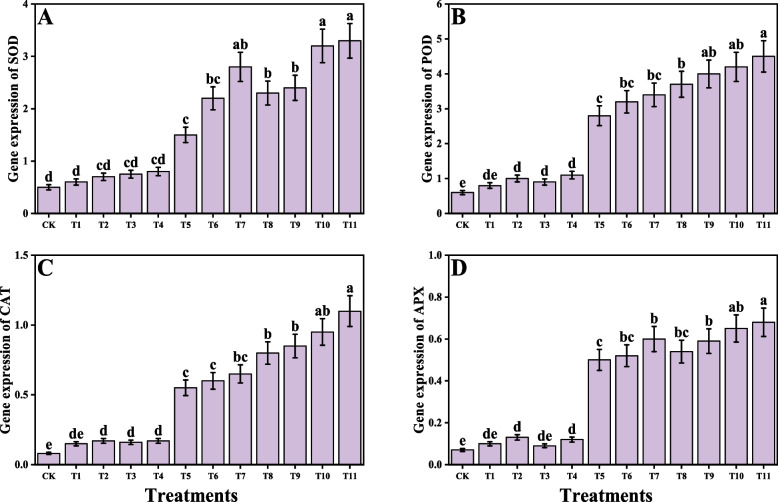
Effect of alone and/or combinatorial application of *Serratia marcescens and Pseudomonas fluorescens* on relevant gene expression i.e., superoxidase dismutase (**A**), peroxidase (**B**), catalase (**C**) and ascorbate peroxidase (**D**) of rice (*Oryza sativa* L.) grown under the cadmium stress (100 µM) in the soil. Values in the figures indicate just one harvest of four replications. Mean ± SD (*n* = 4). One-way ANOVA was performed and mean differences were tested by HSD (*p* ≤ 0.05). Different lowercase letters on the error bars indicate significant differences between the treatments. The treatments abbreviations are as following: CK: control (no Cd + *S. marcescens* + *P. fluorescens*), T1: *S. marcescens* (10 ppm), T2: *S. marcescens* (20 ppm), T3: *P. fluorescens* (10 ppm), T4: *P. fluorescens* (20 ppm), T5: Cd (100 μM), T6: Cd (100 μM) + *S. marcescens* (10 ppm), T7: Cd (100 μM) + *S. marcescens* (20 ppm), T8: Cd (100 μM) + *P. fluorescens* (10 ppm), T9: Cd (100 μM) + *P. fluorescens* (20 ppm), T10: Cd (100 μM) + *S. marcescens* (10 ppm) + *P. fluorescens* (10 ppm), and T11: Cd (100 μM) + *S. marcescens* (20 ppm) *P. fluorescens* (20 ppm)

**Fig. 6:**
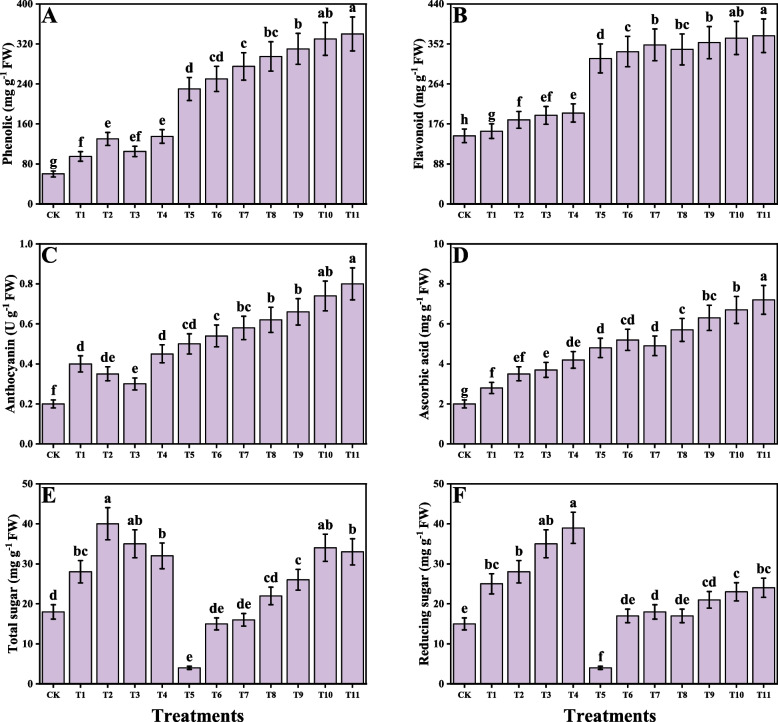
Effect of alone and/or combinatorial application of *Serratia marcescens and Pseudomonas fluorescens* on non-enzymatic compounds and sugar content i.e., phenolic (**A**), flavonoid (**B**), anthocyanin (**C**), ascorbic acid (**D**), total sugar (**E**) and reducing sugar (**F**) of rice (*Oryza sativa* L.) grown under the cadmium stress (100 µM) in the soil. Values in the figures indicate just one harvest of four replications. Mean ± SD (*n* = 4). One-way ANOVA was performed and mean differences were tested by HSD (*p* ≤ 0.05). Different lowercase letters on the error bars indicate significant differences between the treatments. The treatments abbreviations are as following: CK: control (no Cd + *S. marcescens* + *P. fluorescens*), T1: *S. marcescens* (10 ppm), T2: *S. marcescens* (20 ppm), T3: *P. fluorescens* (10 ppm), T4: *P. fluorescens* (20 ppm), T5: Cd (100 μM), T6: Cd (100 μM) + *S. marcescens* (10 ppm), T7: Cd (100 μM) + *S. marcescens* (20 ppm), T8: Cd (100 μM) + *P. fluorescens* (10 ppm), T9: Cd (100 μM) + *P. fluorescens* (20 ppm), T10: Cd (100 μM) + *S. marcescens* (10 ppm) + *P. fluorescens* (10 ppm), and T11: Cd (100 μM) + *S. marcescens* (20 ppm) *P. fluorescens* (20 ppm)

In the present study different oxidative stress biomarkers i.e., malondialdehyde (MDA), hydrogen peroxide (H_2_O_2_) were measured from *O. sativa* as presented in Fig. 4. According to the results, it was observed that the Cd stress caused a significant increase in MDA and H_2_O_2_ contents compared to the control. Although the application of *S. marcescens* and *P. fluorescens* decreases the MDA and H_2_O_2_ contents in *O. sativa*. We have also noticed that the individual application of *S. marcescens* and *P. fluorescens* also decreases MDA and H_2_O_2_ content, when plants were grown without the contamination of Cd in the soil. In addition, combined application of *S. marcescens* and *P. fluorescens* showed more severe results when compared to the plants which grown in the alone application of *S. marcescens* and *P. fluorescens*. Different enzymatic antioxidants i.e., superoxidase dismutase (SOD), ascorbate peroxidase (APX), peroxidase (POD), catalase (CAT) and nonenzymatic compounds i.e., total phenolics, flavonoids, ascorbic acid, anthocyanins, total sugar, and reducing sugar and also their relevant gene expression i.e., SOD, POD, CAT and APX were also measured from the tissues of *O. sativa*. The results regarding the enzymatic antioxidants are presented in Fig. 4, while the results regarding their relevant gene expression are presented in Fig. 5 and the non-enzymatic compounds are presented in Fig. 6. According to the results, we have noticed that the Cd stress in the soil significantly increases the enzymatic antioxidants i.e., SOD, POD, CAT and APX and their relevant gene expression and also the non-enzymatic compounds i.e., total phenolics, flavonoids, ascorbic acid, anthocyanins, total sugar, and reducing sugar compared to the plants grown in the control treatment. Present findings also showed that the alone application of either *S. marcescens* and *P. fluorescens* also increases the activity of SOD, POD, CAT and APX and their relevant gene expression and also the non-enzymatic compounds i.e., total phenolics, flavonoids, ascorbic acid, anthocyanins, total sugar, and reducing sugar compared to the plants which were not treatment with the application of *S. marcescens* and *P. fluorescens* in Cd stressed soil. In addition, the maximum activity of SOD, POD, CAT and APX and their relevant gene expression and also the nonenzymatic compounds i.e., total phenolics, flavonoids, ascorbic acid, anthocyanins, total sugar, and reducing sugar were observed in the plants which grown in the combined application of *S. marcescens* and *P. fluorescens* application. Cd concentration of the roots and shoots were also measured in the present study when *O. sativa* grown under the Cd stressed soil with the individual or combined application of *S. marcescens* and *P. fluorescens*. The data regarding the Cd concentration from the roots and shoots are presented in Fig. 4. According to the results we have noticed that the Cd concentration in the soil medium significantly increased the Cd concentration in the roots and shoots when compared to the control. The application of *S. marcescens* and *P. fluorescens* further increased the concentration of Cd in the roots and shoots when compared to the plants which were grown in the Cd-treated plants without the application of *S. marcescens* and *P. fluorescens*.

### Impact of individual/or combinatorial application of PGPR on AsA − GSH cycle, proline metabolism and cellular fractionation under Cd Stress

**Fig. 7:**
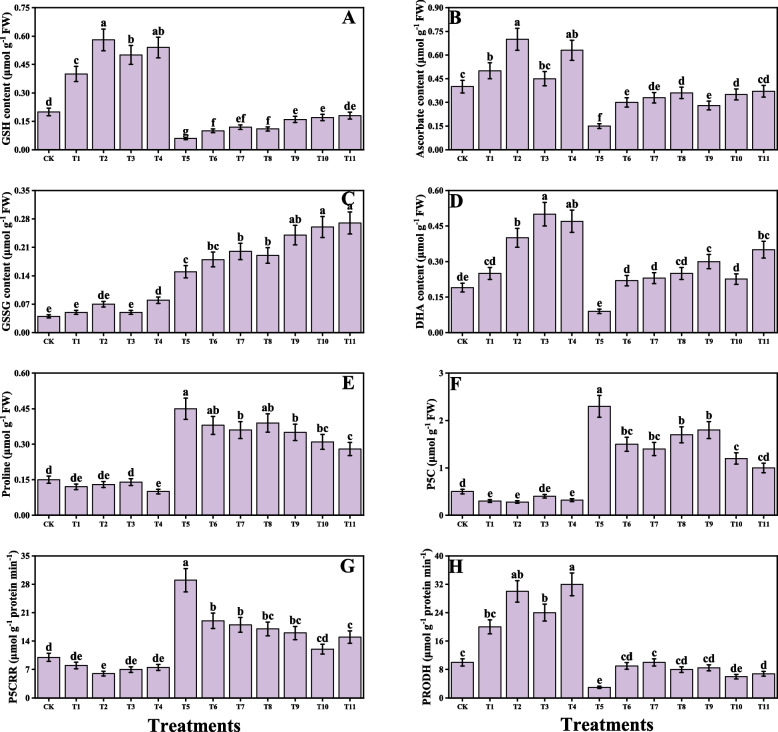
Effect of alone and/or combinatorial application of *Serratia marcescens and Pseudomonas fluorescens* on on glutathione (**A**), ascorbate (**B**), glutathione disulfide (**C**), dehydroascorbic acid (**D**), proline (**E**), pyrroline-5-carboxylate (**F**), pyrroline-5-carboxylate reductase (**G**) and pyrroline-5- carboxylate dehydrogenase (**H**) of rice (*Oryza sativa* L.) grown under the cadmium stress (100 µM) in the soil. Values in the figures indicate just one harvest of four replications. Mean ± SD (*n* = 4). One-way ANOVA was performed and mean differences were tested by HSD (*p* ≤ 0.05). Different lowercase letters on the error bars indicate significant differences between the treatments. The treatments abbreviations are as following: CK: control (no Cd + *S. marcescens* + *P. fluorescens*), T1: *S. marcescens* (10 ppm), T2: *S. marcescens* (20 ppm), T3: *P. fluorescens* (10 ppm), T4: *P. fluorescens* (20 ppm), T5: Cd (100 μM), T6: Cd (100 μM) + *S. marcescens* (10 ppm), T7: Cd (100 μM) + *S. marcescens* (20 ppm), T8: Cd (100 μM) + *P. fluorescens* (10 ppm), T9: Cd (100 μM) + *P. fluorescens* (20 ppm), T10: Cd (100 μM) + *S. marcescens* (10 ppm) + *P. fluorescens* (10 ppm), and T11: Cd (100 μM) + *S. marcescens* (20 ppm) *P. fluorescens* (20 ppm)

**Fig. 8:**
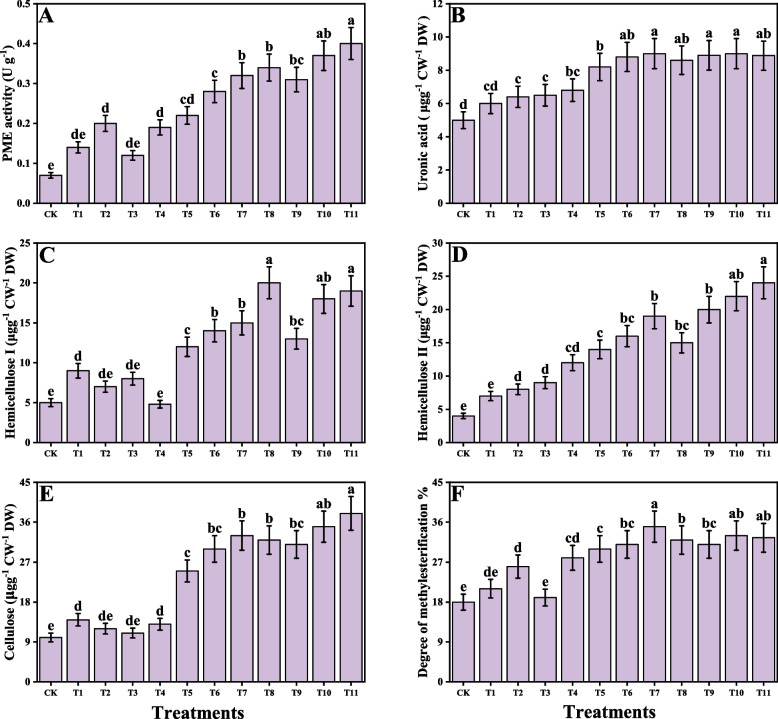
Effect of alone and/or combinatorial application of *Serratia marcescens and Pseudomonas fluorescens* on cellular fractionation i.e., pectin methylesterase activity (**A**), uronic acid (**B**), hemicellulose I (**C**), hemicellulose II (**D**), cellulose (**E**) and pectin methylesterase (**F**) of rice (*Oryza sativa* L.) grown under the cadmium stress (100 µM) in the soil. Values in the figures indicate just one harvest of four replications. Mean ± SD (*n* = 4). One-way ANOVA was performed and mean differences were tested by HSD (*p* ≤ 0.05). Different lowercase letters on the error bars indicate significant differences between the treatments. The treatments abbreviations are as following: CK: control (no Cd + *S. marcescens* + *P. fluorescens*), T1: *S. marcescens* (10 ppm), T2: *S. marcescens* (20 ppm), T3: *P. fluorescens* (10 ppm), T4: *P. fluorescens* (20 ppm), T5: Cd (100 μM), T6: Cd (100 μM) + *S. marcescens* (10 ppm), T7: Cd (100 μM) + *S. marcescens* (20 ppm), T8: Cd (100 μM) + *P. fluorescens* (10 ppm), T9: Cd (100 μM) + *P. fluorescens* (20 ppm), T10: Cd (100 μM) + *S. marcescens* (10 ppm) + *P. fluorescens* (10 ppm), and T11: Cd (100 μM) + *S. marcescens* (20 ppm) *P. fluorescens* (20 ppm)

In the present study, proline-related attributes and AsA–GSH cycle were also measured from the *O. sativa* under the Cd stress. The proline-related attributes and AsA–GSH cycle are presented in Fig. 7. The proline-related parameters such as proline, pyrroline5-carboxylate, pyrroline-5-carboxylate reductase, and pyrroline-5-carboxylate dehydrogenase were also measured from the *O. sativa* tissue, and results showed that the Cd toxicity induced a significant decrease in the proline, pyrroline-5-carboxylate, and pyrroline5-carboxylate reductase compared to the control except the pyrroline-5-carboxylate dehydrogenase. However, the application of *S. marcescens* and *P. fluorescens* either individually or in combined form induced a significant decrease in the content of proline, pyrroline-5-carboxylate, and pyrroline-5-carboxylate reductase, compared to those plants which were grown without the application of *S. marcescens* and *P. fluorescens* except the pyrroline-5-carboxylate dehydrogenase. AsA–GSH cycle including the contents of glutathione, ascorbate, glutathione disulfide, and dehydroascorbic acid was also measured, and we have noticed that the Cd toxicity significantly decreases the content of glutathione, ascorbate, and dehydroascorbic acid while increases the content of glutathione disulfide from the tissues of the *O. sativa* (Fig. 7). However, the application of *S. marcescens* and *P. fluorescens* increases the contents of glutathione, ascorbate, and dehydroascorbic acid compared to the plants which were grown without the application of *S. marcescens* and *P. fluorescens*. Cellular compartment fractionation, i.e., pectin methylesterase activity, uronic acid, hemicellulose I, hemicellulose II, cellulose, and pectin methylesterase was also determined from the *O. sativa* and is presented as Fig. 8. Results from the present study showed that the Cd toxicity causes a significant increase in the pectin methylesterase activity, uronic acid, hemicellulose I, hemicellulose II, cellulose, and pectin methylesterase when compared to the control. However, the application of *S. marcescens* and *P. fluorescens* either individually or in combined form further increases the content of pectin methylesterase activity, uronic acid, hemicellulose I, hemicellulose II, cellulose, and pectin methylesterase in *O. sativa*.

## Discussion

Experimental findings confirm that *S. marcescens* effectively mitigates the adverse effects of Cd toxicity in plants by employing a range of physiological, biochemical, and molecular defense mechanisms. Under Cd stress, *S. marcescens* significantly enhances chlorophyll-a, chlorophyll-b, total chlorophyll, carotenoid content, stomatal conductance, net photosynthesis, transpiration rate, and intercellular CO₂ by modulating the pathways responsible for chlorophyll biosynthesis and degradation, thereby improving overall photosynthetic efficiency [15]. In terms of the AsA–GSH cycle, *S. marcescens* plays a pivotal role by elevating the concentrations of key redox-regulating molecules. These include reduced glutathione, ascorbic acid, glutathione disulfide and dehydroascorbic acid, all of which contribute to the detoxification of reactive oxygen species (ROS) and maintenance of cellular redox homeostasis [33]. Regarding cellular fractionation, *S. marcescens* contributes to the stabilization of cell wall components, such as pectin methylesterase activity, uronic acid, hemicellulose I, hemicellulose II, cellulose, and pectin methylesterase, thereby preserving cellular structure and integrity under metal-induced oxidative stress [34]. For proline-related metabolism, the bacterium enhances proline accumulation such as proline, pyrroline-5-carboxylate, and pyrroline5-carboxylate reductase and pyrroline-5-carboxylate dehydrogenase, a known osmoprotectant, which stabilizes proteins and membranes and supports osmotic adjustment under stress conditions [12]. Antioxidant defense mechanisms are significantly upregulated with *S. marcescens* treatment. Activities of key antioxidant enzymes such as SOD, POD, CAT and APX are markedly increased. This enzymatic response effectively reduces lipid peroxidation, as indicated by lower MDA and H_2_O_2_ content, and protects cellular organelles from oxidative injury [35]. Gene expression analysis reveals that *S. marcescens* activates multiple stress-responsive genes, including those associated with antioxidant defense (e.g., *Fe-SOD*, *CAT*, *POD*, and *APX*), metal transport, and stress signaling, thereby strengthening the plant’s intrinsic defense system against Cd toxicity [15]. In the context of non-enzymatic stress responses, *S. marcescens* enhances the levels of total phenolics, flavonoids, ascorbic acid, anthocyanins, total sugar, and reducing sugar, which function as osmolytes and metabolic energy sources. These sugars help maintain osmotic balance and provide essential carbon skeletons for stress-induced metabolic processes [10]. Regarding Cd uptake, *S. marcescens* significantly limits the accumulation of Cd in plant tissues by possibly altering root exudation patterns, chelating heavy metals in the rhizosphere, or influencing metal transporter activity. This ultimately contributes to reduced translocation of Cd from roots to shoots, improving plant health and biomass accumulation [33].

Our findings are consistent with earlier studies highlighting the crucial role of *P. fluorescens* in enhancing plant tolerance to Cd stress through diverse physiological, biochemical, and molecular mechanisms. Under Cd-contaminated conditions, *P. fluorescens* significantly improves chlorophyll-a, chlorophyll-b, total chlorophyll, carotenoid content, stomatal conductance, net photosynthesis, transpiration rate, and intercellular CO₂, thereby supporting photosynthetic efficiency and maintaining energy production required for stress response and recovery [36]. In the context of the AsA–GSH cycle, *P. fluorescens* enhances the synthesis of critical antioxidants such as ascorbic acid, dehydroascorbic acid DHA, reduced glutathione, and glutathione disulfide, which are essential in neutralizing ROS and maintaining cellular redox homeostasis [14]. Cellular fractionation is improved by the bacterium through reinforcement of structural components such as pectin methylesterase activity, uronic acid, hemicellulose I, hemicellulose II, cellulose, and pectin methylesterase in the cell wall, thereby reducing Cd-induced disruption of cell architecture and maintaining structural integrity [37]. With respect to proline metabolism, *P. fluorescens* significantly increases the accumulation of free proline, which acts as an osmoprotectant, stabilizes proteins and membranes, and contributes to ROS detoxification [38]. The bacterium also strengthens the antioxidant defense system, significantly increasing the enzymatic activities of SOD, POD, CAT and APX. These enzymes collectively reduce oxidative damage by lowering MDA and H₂O₂ contents, thus protecting membrane-bound organelles from peroxidation and structural breakdown [39]. At the molecular level, gene expression analysis indicates that *P. fluorescens* upregulates stress-related genes, including those involved in antioxidant defense and metal detoxification pathways (e.g., *SOD1*, *CAT1*, *APX1*, *PCS1*), thereby enhancing the plant’s intrinsic capacity to withstand Cd-induced stress [40]. Furthermore, *P. fluorescens* enhances non-enzymatic stress mitigation by increasing total and reducing sugar concentrations, which contribute to osmotic adjustment and supply metabolic energy essential for cellular maintenance and repair. Importantly, *P. fluorescens* significantly reduces Cd uptake and translocation by limiting Cd mobility in the rhizosphere, altering metal transporter expression, or promoting metal chelation, thereby reducing the toxic burden within plant tissues [41]. This reduction in Cd accumulation is reflected in improved shoot length, shoot fresh weight, root length, root fresh weight, shoot dry weight, root dry weight, and overall plant growth [42]. Collectively, these multifactorial responses underscore the effectiveness of *P. fluorescens* in alleviating Cd toxicity and affirm its utility as a potent bioinoculant for enhancing plant yield and productivity under heavy metal stress.

The combined application of *S. marcescens* and *P. fluorescens* demonstrated a synergistic effect that more effectively alleviated Cd stress in *O. sativa* compared to individual applications. This synergism significantly increased chlorophyll-a, chlorophyll-b, total chlorophyll, carotenoid content, stomatal conductance, net photosynthesis, transpiration rate, and intercellular CO₂, thereby optimizing photosynthetic performance and energy capture more efficiently than either strain alone [10]. Activation of the AsA–GSH cycle was markedly intensified under the combined treatment, resulting in elevated concentrations of glutathione, glutathione disulfide, ascorbic acid, and dehydroascorbic acid. This enhanced antioxidant pool contributed to a stronger redox buffering capacity and more effective detoxification of ROS [12]. Cellular fractionation was also better preserved due to improved stabilization of cell wall components such as pectin methylesterase activity, uronic acid, hemicellulose I, hemicellulose II, cellulose, and pectin methylesterase, thereby maintaining cellular structure and integrity under Cd-induced stress [43]. In terms of proline metabolism, the combined bacterial treatment significantly increased proline accumulation, supporting osmotic adjustment and protecting cellular proteins and membranes from osmotic imbalance and oxidative injury [44]. The combined inoculation also led to a notable upregulation in the activities of major antioxidant enzymes, including SOD, POD, CAT and APX. These enhancements were accompanied by marked reductions in MDA and H₂O₂ contents, indicating decreased lipid peroxidation and less oxidative damage to membrane-bound organelles [45]. Gene expression analysis further confirmed that co-application of *S. marcescens* and *P. fluorescens* resulted in greater upregulation of stress-responsive genes associated with antioxidant defense, metal detoxification, and signaling pathways, leading to a more robust plant defense system [14]. Crucially, the co-application led to a more substantial reduction in Cd uptake and translocation to aboveground tissues, thereby decreasing the overall Cd burden in the plant and promoting healthier growth, greater shoot and root biomass, and improved stress tolerance [15]. Collectively, these findings emphasize the superior efficacy of using a combined PGPR approach as a sustainable and environmentally friendly strategy for enhancing plant resilience, physiological functioning, and productivity in metal-contaminated agricultural systems.

## Conclusion

The results of this study reveal the effects of *S. marcescens* and *P. fluorescens* application on Cd-stressed *O. sativa*. We demonstrated that *O*. *sativa* is a metal-tolerant species, a characteristic conferred by its active antioxidant defense system. The application of *S. marcescens* and *P. fluorescens* (individually or in combination) to cadmium-stressed plants improved plant growth and biomass, photosynthetic pigment levels, gas exchange attributes, sugars, ascorbate–glutathione cycle, cellular fractionation, and proline metabolism while alleviating oxidative stress and reducing oxidative stress in 60-day-old *Oryza sativa*. Furthermore, combined application of *S. marcescens* and *P. fluorescens* showed more severe results and enhance plant tolerance compared to the single application. This is an initial investigation, and more research using different species in this field will be needed to identify the ideal dosages of various phytohormones in single and combination forms.

## Supplementary Information

Supplementary Material 1

## Data Availability

The data that support the findings of this study are available from the corresponding author upon reasonable request.
