# Supplementary material for: Eco-friendly role of serratia marcescens and pseudomonas fluorescens in enhancing rice growth and mitigating cadmium toxicity via uptake modulation and antioxidant regulation
Source: BMC Plant Biol. 2025 May 28;25:718. doi: 10.1186/s12870-025-06693-6 (PMC12117749; doi:10.1186/s12870-025-06693-6)
Supplement: Supplementary file 1 — Supplementary Material 1 [file 12870_2025_6693_MOESM1_ESM.docx]

**Table S1.** Gene-specific primers sequences used in present study

| Gene | Primer Sequence (5′-3′) | Gene Accession Number |
| --- | --- | --- |
| *Fe-SOD* | F: ATCTTAGTTATGGTTCTCTTTGT  R: ATGGTGTAGAGCCTTTTCATAT | M64267 |
| *POD* | F: TTGAAATAAAC CAAAGGAGTAGT  R: AATAATTATTTGAATCTCTTTAAGG | AF145349 |
| *CAT* | F: AGCATCTCACCTGAACTTGAA  R: AGGTGAGAGGTTTGTGGCC | AF035252 |
| *APX* | F: CGTGACGATGATTGGGAAGT  R: TGATAGTGATCTTTCGGACCT | NM_001354113 |
